# Impact of (intestinal) LAL deficiency on lipid metabolism and macrophage infiltration

**DOI:** 10.1016/j.molmet.2023.101737

**Published:** 2023-05-12

**Authors:** Valentina Bianco, Melanie Korbelius, Nemanja Vujic, Alena Akhmetshina, Melina Amor, Dagmar Kolb, Anita Pirchheim, Ivan Bradic, Katharina B. Kuentzel, Martin Buerger, Silvia Schauer, Huyen T.T. Phan, Dominik Bulfon, Gerald Hoefler, Robert Zimmermann, Dagmar Kratky

**Affiliations:** 1 Gottfried Schatz Research Center, Molecular Biology and Biochemistry, Medical University of Graz, Graz, Austria; 2 Core Facility Ultrastructure Analysis, Center for Medical Research, Medical University of Graz, Graz, Austria; 3 BioTechMed-Graz, Graz, Austria; 4 Diagnostics and Research Institute of Pathology, Medical University of Graz, Graz, Austria; 5 Institute of Molecular Biosciences, University of Graz, Graz, Austria

**Keywords:** Lysosomal acid lipase, LAL-D, Small intestine, Enterocytes, Intestinal lipid absorption, Macrophages

## Abstract

**Objective:**

To date, the only enzyme known to be responsible for the hydrolysis of cholesteryl esters and triacylglycerols in the lysosome at acidic pH is lysosomal acid lipase (LAL). Lipid malabsorption in the small intestine (SI), accompanied by macrophage infiltration, is one of the most common pathological features of LAL deficiency. However, the exact role of LAL in intestinal lipid metabolism is still unknown.

**Methods:**

We collected three parts of the SI (duodenum, jejunum, ileum) from mice with a global (LAL KO) or intestine-specific deletion of LAL (iLAL KO) and corresponding controls.

**Results:**

We observed infiltration of lipid-associated macrophages into the lamina propria, where neutral lipids accumulate massively in the SI of LAL KO mice. In addition, LAL KO mice absorb less dietary lipids but have accelerated basolateral lipid uptake, secrete fewer chylomicrons, and have increased fecal lipid loss. Inflammatory markers and genes involved in lipid metabolism were overexpressed in the duodenum of old but not in younger LAL KO mice. Despite the significant reduction of LAL activity in enterocytes of enterocyte-specific (iLAL) KO mice, villous morphology, intestinal lipid concentrations, expression of lipid transporters and inflammatory genes, as well as lipoprotein secretion were comparable to control mice.

**Conclusions:**

We conclude that loss of LAL only in enterocytes is insufficient to cause lipid deposition in the SI, suggesting that infiltrating macrophages are the key players in this process.

## Abbreviations

LAL: lysosomal acid lipase
SI: small intestine
LAL KO: global LAL deficiency
iLAL KO: enterocyte-specific LAL deficiency
CE: cholesteryl ester
TG: triacylglycerol
FA: fatty acid
FC: free cholesterol
CM: chylomicron

## 1 Introduction

Absorption of dietary lipids in the intestine is a fundamental step of whole-body lipid homeostasis. Complex dietary lipids, mainly triacylglycerols (TGs), phospholipids (PLs) and cholesteryl esters (CEs), are already metabolized in the mouth and further in the stomach by various lipases [1] before they are finally degraded in the proximal duodenum by mixing with pancreatic and biliary secretions. TGs are hydrolyzed in the intestinal lumen by pancreatic lipase to fatty acids (FAs) and monoacylglycerol (MG) [2], whereas CE degradation is catalyzed by pancreatic cholesterol esterase to release free cholesterol (FC) and FAs [3]. These lipids are then emulsified with bile acids and PLs to form micelles [4], which facilitate the uptake of lipids through the apical side of enterocytes either by passive diffusion or protein-mediated transport [5]. Among them, cluster of differentiation 36 (CD36) is the major transporter for FAs [6], whereas Niemann-Pick C1-like 1 (NPC1L1) has been identified as the main transport protein for cholesterol [7]. After absorption, FAs, MGs, and FCs are shuttled to the endoplasmic reticulum (ER) [8] and re-esterified into TGs and CEs to prevent lipotoxicity. Due to their high hydrophobicity, these newly formed lipids must be stabilized in the hydrophilic environment by sequestration/association with amphipathic molecules. Therefore, lipids destined for secretion fuse with apolipoprotein B48 (ApoB48) molecules to form chylomicrons (CMs), which are then delivered to peripheral tissues via the lymphatics [9]. In addition, lipids may also be temporarily stored in cytosolic lipid droplets (cLDs) and mobilized upon need to be further secreted in the form of CMs [10].

TGs stored within cLDs are catabolized either by neutral lipolysis in the cytoplasm, initiated by adipose triglyceride lipase (ATGL) and completed by hormone-sensitive lipase (HSL) and monoglyceride lipase (MGL), or by lipophagy [11]. The latter is a process in which a cLD or a part of a cLD is engulfed by a double-membrane autophagosome, which then fuses with a lysosome to form an autolysosome [12]. In this organelle, lysosomal acid lipase (LAL) is the only enzyme known to hydrolyze TGs, diacylglycerols (DGs), CEs, and retinyl esters to release FAs, MGs, FC, and retinol, which are then secreted or used by the cell for energy production, membrane assembly, steroidogenesis, or signaling purposes [13–15]. This process also occurs in enterocytes, where nascent LDs are scavenged from the ER just minutes after absorption of dietary lipids and transported to the lysosome for lipid degradation [16]. This postprandial process must be tightly regulated as elevated postprandial lipids can lead to dyslipidemia and obesity. Paradoxically, lipophagy is induced in enterocytes postpran-dially via a pathway involving fibroblast growth factor (FGF) 15/19, small heterodimer partner (SHP), and transcription factor EB (TFEB), preventing excessive secretion of lipids into the bloodstream [17].

Given the important role of the lysosome in lipid metabolism, LAL has become the focus of numerous studies. Mutations in the LAL-encoding LIPA gene are responsible for the development of LAL deficiency (LAL-D), which is characterized by the accumulation of CEs and TGs predominantly in hepatocytes, adrenal glands, small intestine (SI), and macrophages [18]. Depending on the LIPA mutation and residual LAL activity, LAL-D with <1% residual LAL activity may cause LAL-D in infancy (formerly known as Wolman disease), which leads to death within the first 3–6 months of life. Patients suffer from hepatomegaly, liver damage, diarrhea, vomiting, intestinal malabsorption, and failure to thrive [19]. Partial LAL-D with up to 10% residual lipase activity leads to later-onset LAL-D (formerly named CE storage disease) in childhood or even adulthood. These patients present with symptoms such as hepatomegaly, dyslipidaemia, and cardiovascular complications [19]. In addition to dyslipidemia and the abundance of lipid-filled lysosomes in various cells and tissues, one of the most common symptoms of LAL-D patients is lipid malabsorption throughout the SI, frequently resulting in severe steatorrhea. Moreover, massive infiltration of macrophages in the lamina propria of the SI and colon is visible in LAL-D patients, causing a club-shaped structure of the villi and intestinal inflammation [20].

To further investigate the role of LAL in intestinal lipid metabolism, we used global LAL-deficient (LAL KO) mice and mice lacking the enzyme exclusively in enterocytes (iLAL KO). Our results demonstrate that loss of LAL solely in enterocytes is not a sufficient trigger to replicate the severe intestinal phenotype observed in global LAL KO mice.

## 2 Materials And Methods

### 2.1 Animals

To generate enterocyte-specific LAL (iLAL KO) mice, we crossed mice carrying a LoxP-modified Lipa allele [21] with transgenic mice expressing Cre recombinase under the control of the intestinal epithelial cell-specific villin promoter [22]. Age- and sex-matched wild type (WT), LAL KO, LAL^flox/flox^ and iLAL KO mice (all on the C57BL/6J background) were housed in a clean and temperature-controlled environment (22 ± 1 °C; relative humidity, 45%–65%) with unlimited access to food and water on a regular 12 h/12 h light–dark cycle. Mice were fed either a standard chow diet (11.9% caloric intake from fat; Altromin, Lage, Germany) or a high fat/high cholesterol diet (HF/HCD; 34% crude fat, 1% cholesterol; Ssniff®, Soest, Germany) for the indicated time periods. All experiments were performed in accordance with the European Directive 2010/63/EU and approved by the Austrian Federal Ministry of Education, Science and Research (Vienna, Austria; BMWFW-66.010/0109-WF/V/3b/2015, 2020-0.129.904).

### 2.2 Plasma and tissue lipid analysis

TGs, total cholesterol (TC), FCs, and CEs from plasma and the three parts of the SI (duodenum, jejunum, ileum) were isolated and measured as previously described [23].

### 2.3 Energy metabolism in vivo

Energy intake and energy expenditure were determined using a climate-controlled indirect calorimetry system (TSE Systems, Bad Homburg, Germany). Chow diet or 16-week HF/HCD-fed control and iLAL KO mice were housed in metabolic cages in a 12 h/12 h light–dark cycle with free access to food and water. Energy expenditure and respiratory exchange ratio were measured every 15 min.

### 2.4 Histology, immunohistochemistry (IHC), and oil red O (ORO) staining

SIs were fixed in 4% neutral-buffered formalin for 24 h and then stored in 30% sucrose until cryosections (7 μm) were cut (HM 560 Cryo-Star; Microm International GmbH, Walldorf, Germany) and subjected to routine haematoxylin and eosin staining [24].

For CD68 IHC, SIs were fixed in 4% neutral-buffered formalin for 24 h and then stored in PBS at 4 °C until paraffin embedding. Sections (7 mm) were deparaffinized, and staining was performed using a Leica Bond MAX immunostainer (Leica Microsystems, Wetzlar, Germany) and CD68 antibody (1:1,000; Cell Signaling Technologies, Danvers, MA). To visualize neutral lipids and nuclei, cryosections (7 mm) were stained with ORO (Sigma-Aldrich, St. Louis, MO) and DAPI (Akoya Bioscience, Marlborough, MA), respectively. Images were acquired at 60 × magnification (Apo 60 × Oil λS objective) using a Nikon Eclipse T*i* microscope (NIKON Corporation, Tokyo, Japan) equipped with a Nikon A1 camera.

### 2.5 CM secretion

Lipid absorption and subsequent CM secretion were determined as previously described [25] with minor modifications. Briefly, 4-h fasted mice were injected with 500 mg/kg poloxamer-P407 (Sigma-Aldrich, St. Louis, MO) and 30 min post-injection, gavaged with 100 ml corn oil containing 2 μCi [^3^H]-triolein (Perkin Elmer, Waltham, MA), 0.5 μCi [^14^C]-cholesterol (ARC Inc, St. Louis, MO), and 0.25% cholesterol. Radioactivity in plasma was measured 1, 2, 3, and 4 h after gavage by liquid scintillation counting.

### 2.6 Isolation of enterocytes

Primary enterocytes were isolated as previously described [26]. Briefly, the intestinal segment was washed with Buffer A (115 mM NaCl, 5.4 mM KCl, 0.96 mM NaH_2_PO_4_, 26.19 mM NaHCO_3_, 5.5 mM glucose). Thereafter, one end of the intestine was tied and the lumen was filled with Buffer B (67.5 mM NaCl, 1.5 mM KCl, 0.96 mM NaH_2_PO_4_, 26.19 mM NaHCO_3_, 27 mM sodium citrate, 5.5 mM glucose). After incubation with 0.9% NaCl for 15 min at 37 °C, the luminal content was discarded and the jejunum was filled with Buffer C (Buffer A plus 1.5 mM EDTA and 0.5 mM DTT). After another incubation with 0.9% NaCl for 10 min at 37 °C, the luminal content was collected, filtered, and centrifuged at 1,500×*g* for 5 min at RT. All buffers were adjusted to pH 7.4 and aerated with 95% O_2_ and 5% CO_2_ before use.

### 2.7 CE and TG hydrolase activity assays

Enterocytes isolated from jejuna were lysed in acid citrate buffer (containing 54% of 100 mM citric acid monohydrate and 46% of 100 mM trisodium citrate, dehydrated, pH 4.2; Carl Roth, Karlsruhe, Germany) as previously described [27]. Briefly, cells were sonicated twice for 10 s on ice, centrifuged at 1,000×*g* and 4 °C for 10 min, and protein concentrations were determined in the supernatant. Thereafter, 50 mg of protein were diluted to a final volume of 100 μl in citrate buffer. We used 0.2 mM cholesteryl oleate/sample, 0.04 μCi/sample cholesteryl [1–^14^C]-oleate (Amersham Biosciences, Piscataway, NJ) and 35.5 μg mixed micelles of phosphatidylcholine and phosphatidylinositol (3:1) as substrate to determine CE hydrolase activity. The substrate for the TG hydrolase activity assay contained 0.3 mM triolein/sample, 0.5 μCi/sample [9,10-^3^H(N)]-triolein (Perkin Elmer, Waltham, MA), and 3.5 μg of above-mentioned mixed micelles. Each substrate contained FA-free BSA (Biowest, Nuaillé, France) at a final concentration of 2% in 100 mM citrate buffer. Samples were incubated for 1 h at 37 °C with the respective substrate in the absence or presence of 30 mM Lalistat 2 (#1234569-09-5, Sigma–Aldrich, St. Louis, MO) to inhibit LAL and any other possible acid lipid hydrolases. The reaction was terminated by the addition of 3.25 ml stop solution (methanol:choroform:heptane, 10:9:7, v:v:v) and 1 ml of 100 mM potassium carbonate (pH 10.5) (all Carl Roth, Karlsruhe, Germany). After vortexing and centrifugation at 3,220×*g* and 4 °C for 15 min, the radioactivity in 1 ml of the upper phase was determined by liquid scintillation counting and the release of FAs was calculated as previously described [28].

### 2.8 Electron microscopy

Duodena, jejuna, and ilea from chow diet-fed LAL KO, iLAL KO and corresponding control mice were collected and processed as previously described [27] after 4 h of fasting. The tissues were fixed in 2% paraformaldehyde/2.5% glutaraldehyde for 2 h, washed, post-fixed in cacodylate buffer/OsO_4_ for 2 h, and afterwards washed in cacodylate buffer. After dehydration, samples were infiltrated (propylene oxide and TAAB embedding resin, pure TAAB embedding resin) for 3 h, placed in TAAB embedding resin (2 × 90 min), transferred into embedding molds, and polymerized (72 h, 60 °C). Ultrathin sections (70 nm) stained with lead citrate and platinum blue (International Bio-Analytical Industries, Inc., Boca Raton, FL) were imaged at 120 kV using a Tecnai G2 transmission electron microscope (FEI, Eindhoven, The Netherlands) with a Gatan ultrascan 1000 CCD camera (–20 °C; Digital Micrograph acquisition software; Gatan, Munich, Germany). Stiched overview images were obtained using the SerialEM application [29].

### 2.9 Bis(monoacylglycero)phosphate (BMP) analysis

SI or intestinal scrapings were transferred to 2 ml safe-lock tubes containing two 5-mm steel beads. Lipids were extracted with a modified version of Matayash et al. [30] using 700 μl methyl tert-butyl ether/methanol (3:1; v/v) containing 500 pmol butylated hydroxytoluene, 1% acetic acid, and 150 pmol internal standard (14:0-14:0 BMP, 14:0-14:0-14:0 hemi-BMP, 17:0-17:0 phosphatidylglycerol (PG); Avanti Polar Lipids, Alabaster, AL) per sample. Samples were homogenized by shaking on a Retsch Tissue Lyser (Qiagen, Venlo, The Netherlands) for 2 × 10 s (30 Hz) and lipids were extracted under constant shaking on a thermomixer at 1,400 rpm for 30 min at RT. After the addition of dH_2_O and further incubation for 10 min at RT, the samples were centrifuged for phase separation at 20,000×*g* for 10 min at RT. The upper organic phase was collected, dried under a stream of N_2_, and dissolved in 200 μl methanol/2-propanol/H_2_O (6:3:1; v/v/v) for UPLC/MS analysis.

Chromatographic separation was performed using an Agilent 1290 Infinity II UHPLC equipped with an ACQUITY UPLC BEH C18 Column (2.1 × 150 mm, 1.7 μm; Waters Corporation, Milford, MA), a flow rate of 0.2 ml/min, an injection volume of 2 μl, a column temperature of 50 °C, and a 30-min linear gradient of mobile phase A (methanol/H_2_O; 8/2, v/v) and mobile phase B (2-propanol/methanol; 8/2, v/v). Both solvents contained 10 mM ammonium acetate, 0.1% formic acid, and 8 μM phosphoric acid. BMP and PG species were detected on an Agilent 6470 triple-quadrupole mass spectrometer with Agilent Jet Stream ESI (Agilent Technologies, Santa Clara, CA) under fast polarity switching with simultaneous analysis in ESI positive and negative modes in a single run, controlled by Agilent MassHunter Acquisition software version 10.1. BMP and PG species were analyzed in dynamic multiple reaction monitoring mode using [M–H]^-^ to FA anions transitions in the negative mode. Positive mode: [MNH_4_]^+^ to [MG-H_2_O]^+^ of the respective MG for BMP and [MNH_4_]^+^ to [DG-H_2_O]^+^ of the respective DG for PG. Unit resolution was used in both MS1 and MS2, and the collision energy was set to 25 V for BMP and 15 V for PG, respectively.

Data processing was performed with Agilent MassHunter quantitative analysis software version 10.1 and Agilent MassHunter qualitative analysis software version 10.0. Data were normalized for recovery, extraction, and ionization efficacy by calculating analyte/internal standard ratios (arbitrary unit, AU) and expressed as AU/mg protein.

### 2.10 Isolation and labeling of VLDL with [^3^H]-triolein

VLDL was isolated from human plasma by gradient ultracentrifugation (280,000×*g* at 15 °C for 24 h) in a fixed-angled rotor by adjusting the plasma density to 1.06 g/l with ~10 g NaCl/200 ml plasma and the addition of EDTA (1 g/l) and NaN_3_ (1 g/l). After centrifugation of 40 ml, the upper phase containing VLDL/LDL was collected, dialyzed with distilled water for 30 min, adjusted to 1.027 g/l with NaCl, and centrifuged again for 24 h at 280,000×*g* and 15 °C. VLDL was collected from the upper phase, dried under a stream of N_2_, and 1.6 mg VLDL was labeled with 8 μCi [^3^H]-triolein (Perkin Elmer, Waltham, MA). Samples were redissolved in ethanol for 2 h at 37 °C and incubated overnight at RT under a stream of N_2_ to prevent oxidation.

### 2.11 Basolateral lipid uptake

Chow diet-fed mice that had fasted for 4 h were anesthetized with isofluorane (1.8 l/min) and injected intravenously with 200 ml [^3^H]-triolein-VLDL. Blood was collected after 30 s and 1 h, and then the mice were sacrificed by cervical dislocation. Duodenum, jejunum, ileum, and liver were collected, lyophilized for 24 h, digested in 1 ml of 1 M NaOH, and radioactivity was measured by liquid scintillation counting.

### 2.12 Fecal lipid extraction

Lipids were extracted from 100 mg of lyophilized feces from chow diet-fed LAL KO, iLAL KO, and the corresponding control mice, and TG and TC concentrations were measured using enzymatic test kits (Triglycerides FS, Cholesterol FS; DiaSys, Holzheim, Germany).

### 2.13 Western blotting analysis

Duodenal samples were lysed with RIPA buffer, and 50 mg of protein was separated by SDS-PAGE and transferred to PVDF membranes to detect CD68 (#97778, 1:1,000) and TREM2 (#76765, 1:500) (both purchased from Cell Signaling Technology, Danvers, MA). Monoclonal anti-mouse β-actin was used as loading control (A5316, 1:10,000; Sigma Aldrich, St. Louis, MO). Secondary HRP-conjugated anti-rabbit (31460, 1:2,500, ThermoFisher Scientific, Waltham, MA) and anti-mouse (P0260, 1:1,000, Dako, Glostrup, Denmark) antibodies were visualized by enhanced chemiluminescence detection on a Chem-iDocTM MP imaging system (Bio-Rad Laboratories, Hercules, CA).

### 2.14 RNA isolation and quantitative real-time PCR

Total RNA from tissues was extracted using TRISureTM reagent according to the manufacturer's protocol (Meridian Bioscience, Cincinnati, OH). Total RNA (2 μg) was reverse transcribed using the High-Capacity cDNA Reverse Transcription Kit (ThermoFisher Scientific, Waltham, MA). Quantitative real-time PCR was performed on a CFX96 Real-Time PCR detection system (Bio-Rad Laboratories, Hercules, CA) using the GoTaq® qPCR Master Mix (Promega, Madison, WI). Samples were analyzed in duplicate and normalized to cyclophilin A mRNA expression as reference gene. Expression profiles and associated statistical parameters were determined by the 2^–ΔΔCT^ method. Primer sequences are listed in Supplementary Table S1.

### 2.15 Statistical analysis

Statistical analyses were performed using GraphPad Prism 5.0 software. Significance was calculated by unpaired Student's t-test for comparison of two groups and two-way ANOVA followed by Bonferroni post hoc test for comparison of multiple groups. Data are presented as mean ± SD. For statistical analysis of mRNA expression, values were calculated using the 2^–ΔΔCT^ method and expressed as mean + SD. The following statistical significance levels were used: *, p < 0.05; **, p ≤ 0.01; ***, p ≤ 0.001.

## 3 Results

### 3.1 Efficient LAL knockout in enterocytes of iLAL KO mice

**Figure 1:**
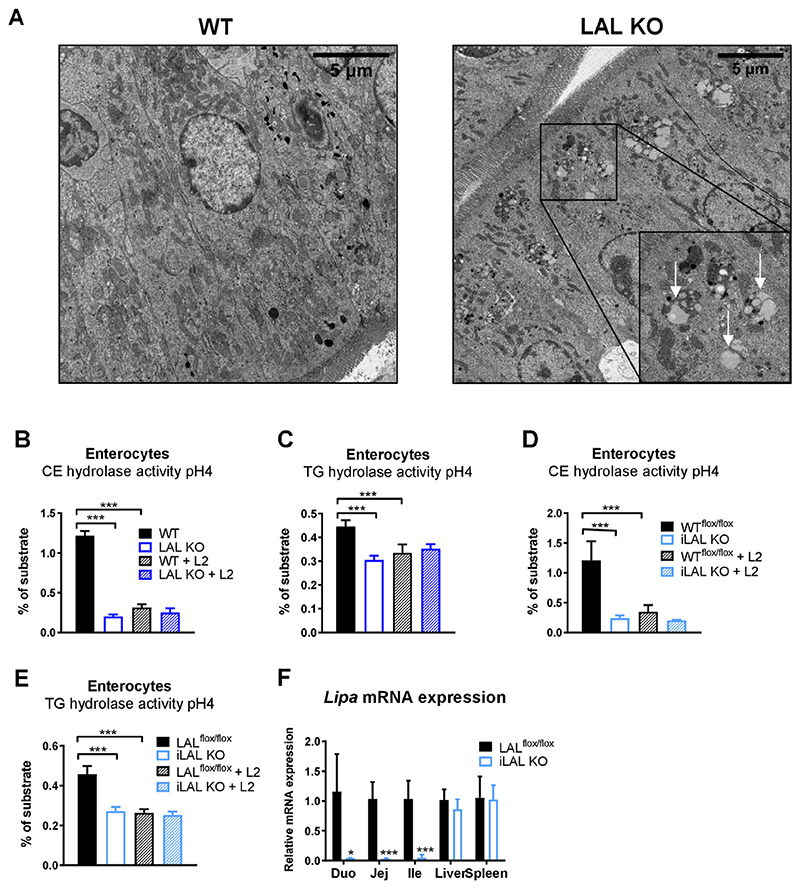
Reduced LAL activity in enterocytes of global LAL KO and iLAL KO mice. **(A)** Electron micrographs of duodenal sections from male WT and LAL KO mice. Scale bar, 5 μm. Arrows indicate lysosomal lipid accumulation. Acid **(B, D)** CE and **(C, E)** TG hydrolase activity in enterocytes isolated from **(B, C)** male LAL KO mice and **(D, E)** female iLAL KO mice and their respective controls. The release of [14C]-FAs was determined by liquid scintillation counting in the absence and presence of Lalistat 2 (L2) (30 μM) (n = 3–6). **(F)** Relative Lipa mRNA expression in the different parts of the SI (duodenum, jejunum, ileum), liver and spleen of female iLAL KO mice (n = 3–5). All mice were fed chow diet and fasted for 4 h before tissue isolation. Data represent mean values + SD; *p < 0.05, ***p ≤ 0.001.

In addition to the previously described prominent lipid accumulation in macrophages of the lamina propria of the SI [31,32], we observed lipid-containing lysosomes in duodenal enterocytes of mice globally lacking LAL (Figure 1A), indicating that enterocyte LAL may play a pivotal role in the metabolism of dietary lipids. Acid CE hydrolase (CEH) and TG hydrolase (TGH) activities, reflecting the enzymatic action of LAL, were reduced by ~ 80% and 30%, respectively, in enterocytes isolated from LAL KO mice (Figure 1B,C). To test the possibility that other enzymes besides LAL may hydrolyze CE at an acidic pH, we included the inhibitor Lalistat 2 (L2) [33] in the assay. L2 failed to further reduce acid CEH activity in enterocyte lysates from LAL KO mice, but efficiently reduced the activity of WT samples to KO levels, suggesting that LAL is the only enzyme in enterocytes that hydrolyzes CE and TG at acidic pH (Figure 1B,C).

To study the impact of intestinal LAL on the observed phenotype, we generated mice lacking LAL exclusively in enterocytes (iLAL KO). *Lipa* mRNA expression that was absent in all three parts of the SI (duodenum, jejunum, ileum) but remained comparable to that of WT mice in the liver and spleen (Figure 1C) confirmed efficient genetic KO exclusively in the SI. Moreover, acid CEH and TGH activity were reduced in enterocytes from iLAL KO mice to a similar extent as in global LAL KO mice (Figure 1E,F).

### 3.2 Enterocyte-specific LAL deficiency does neither affect body weight nor circulating lipid concentrations

**Figure 2:**
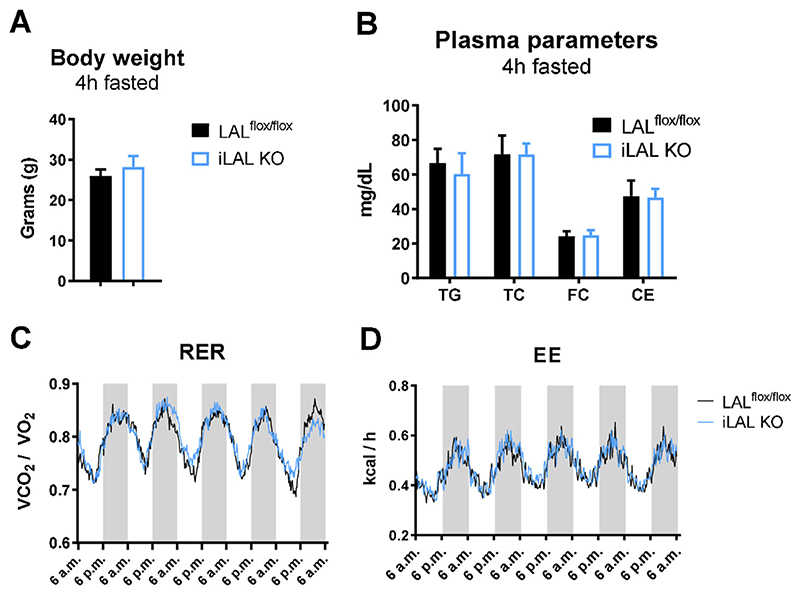
Unchanged body weight, plasma lipid parameters, and energy expenditure in chow diet-fed iLAL KO mice. **(A)** Body weight and **(B)** plasma lipid parameters in chow diet-fed male iLAL KO mice after 4 h of fasting (n = 5–7). **(C)** Respiratory exchange ratio (RER) and **(D)** energy expenditure (EE) of male WT and iLAL KO (n = 5–6). Data represent mean values + SD.

Whereas chow diet-fed global LAL KO mice exhibited decreased body weight and increased plasma cholesterol levels [34], iLAL KO mice had comparable body weight (Figure 2A) and plasma lipid parameters to their control littermates (Figure 2B) when fed chow (Figure 2A,B) or HF/HCD (Supplementary Figs. S1A and B). In contrast to the decreased respiratory exchange ratio (RER) and energy expenditure (EE) in LAL KO mice [35], RER and EE were unaffected in iLAL KO mice fed chow (Figure 2C,D) or HF/HCD (data not shown). Thus, despite the efficient loss of LAL in enterocytes, iLAL KO mice lack phenotypic characteristics of mice with global LAL deficiency.

### 3.3 Massive lipid-rich macrophage infiltration in the lamina propria of LAL KO but not iLAL KO mice

**Figure 3:**
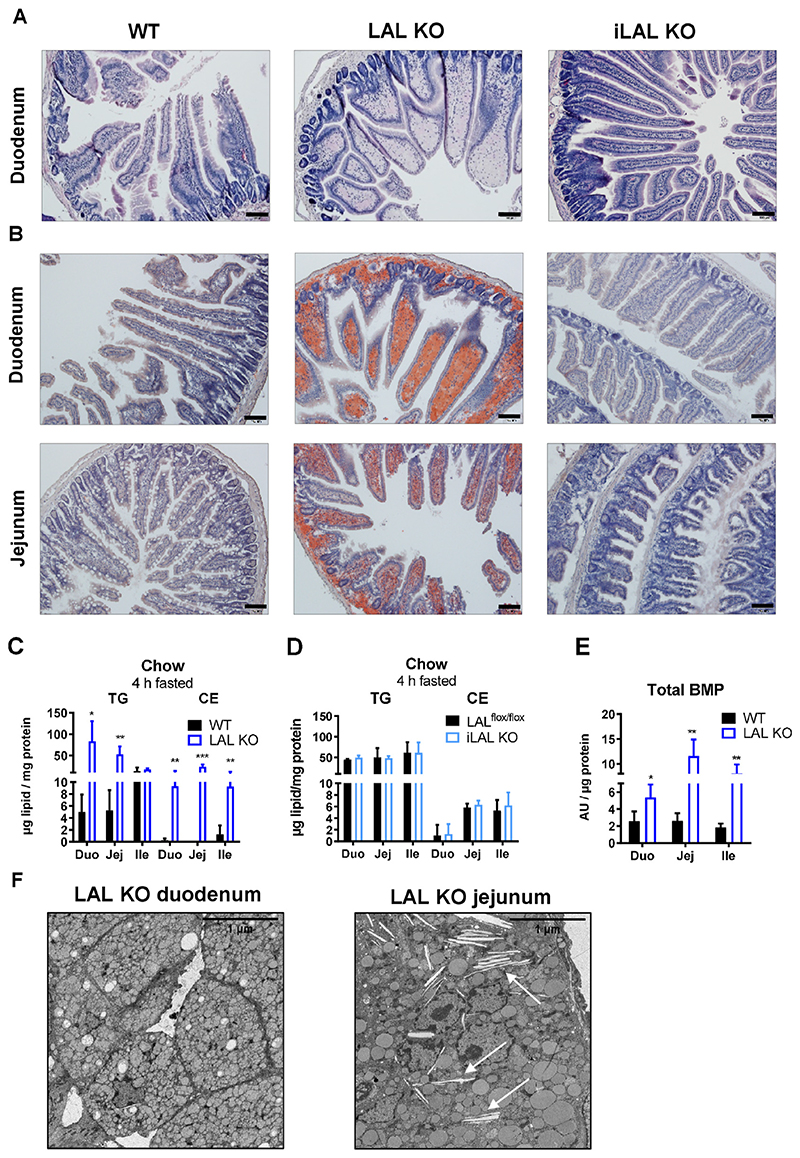
Infiltration of macrophages into the lamina propria results in club-shaped villi in LAL KO mice. **(A)** Representative hematoxylin/eosin staining of duodenal sections from WT, LAL KO, and iLAL KO mice. **(B)** Oil red O staining of duodena and jejuna from 6-h fasted WT, LAL KO, and iLAL KO mice (scale bar, 100 μm). Quantification of intracellular lipids in 4-h fasted **(C)** LAL KO and **(D)** iLAL KO mice. **(E)** BMP quantification in duodena, jejuna, and ilea of LAL KO mice (n = 3-4). **(F)** Electron micrographs of duodenum and jejunum from 4-h fasted male LAL KO mice. Scale bar, 1 μm. Black arrows indicate CE crystals. Data represent mean values (n = 3-5) + SD. *p < 0.05, **p ≤ 0.01, ***p ≤ 0.001.

To gain a better insight into the morphology of the SI, we performed hematoxylin/eosin staining. In contrast to WT mice, the villi of LAL KO duodena displayed a club-shaped structure, which was due to a massive macrophage infiltration in the lamina propria (Figure 3A), which was also visible in the submucosa. However, the morphology of the duodenal villi of iLAL KO mice was comparable to that of WT mice and showed no visible signs of derangement (Figure 3A).

To investigate whether enterocyte LAL could be responsible for the accumulation of TG and CE in the SI of LAL KO mice fed a standard chow diet [32], we stained intestinal sections of all genotypes with ORO to visualize neutral lipids. In LAL KO mice, we observed an accumulation of neutral lipids throughout the SI (duodenum, jejunum, ileum) (Figure 3B, Supplementary Fig. S2A). The staining was most pronounced in the lamina propria and only to a lower extent in enterocytes (Figure 3B, inset), suggesting that the infiltrating macrophages observed by H&E staining (Figure 3A) are the major cell type accumulating neutral lipids in LAL KO SI. In contrast, ORO staining in the SI of iLAL KO mice was comparable to that of controls, indicating unaltered intestinal lipid content (Figure 3B, Supplementary Fig. S2A).

In line with the histological findings, quantification of intracellular lipid content revealed increased TG and CE levels throughout the intestine of LAL KO mice, with the duodenum having the highest TG and the jejunum having the highest CE concentrations (Figure 3C). Intestinal lipid levels in iLAL KO mice were comparable to those of LAL^flox/flox^ mice (Figure 3D).

We next determined BMP levels in LAL KO SI, because this lipid plays a key role in lysosomal integrity and function [36,37]. We found increased BMP concentrations in duodenum, jejunum, and ileum of LAL KO mice (Figure 3E), which is consistent with BMP accumulation in other lysosomal storage diseases [38–40]. To take a closer look at the intestinal lipid accumulation observed in global but not enterocyte-specific LAL KO mice, we examined duodenum and jejunum of 4-h fasted mice by electron microscopy. The LAL KO duodenum showed massive lipid accumulation primarily in the macrophages of the lamina propria (Figure 3F) and only to a lesser extent in the enterocytes, in line with our light microscopic findings. The enlargement of lipid-containing macrophages in the duodenum of LAL KO mice in combination with increased BMP (Figure 3E) suggested that their lysosomes consisted of more and smaller vesicles. In the jejunum of LAL KO mice, infiltrating macrophages did not accumulate as much lipids as in the proximal part of the SI, but we observed the formation of CE crystals, which is consistent with the increased CE deposition in the jejunum of LAL KO mice (Figure 3F). Electron micrographs from WT duodenum and jejunum are presented in Supplementary Figs. S2C and D. The SI of iLAL KO mice showed no differences in their morphology, lysosome or lipid content, and BMP concentrations compared with LAL^flox/flox^ mice (Supplementary Figs. S2D-F).

These findings further indicated that intestinal lipid accumulation was mainly restricted to infiltrating macrophages in the lamina propria of LAL KO mice, independent of enterocyte LAL expression and activity or dietary challenge.

### 3.4 LAL KO mice exhibit impaired dietary but accelerated basolateral lipid uptake

**Figure 4:**
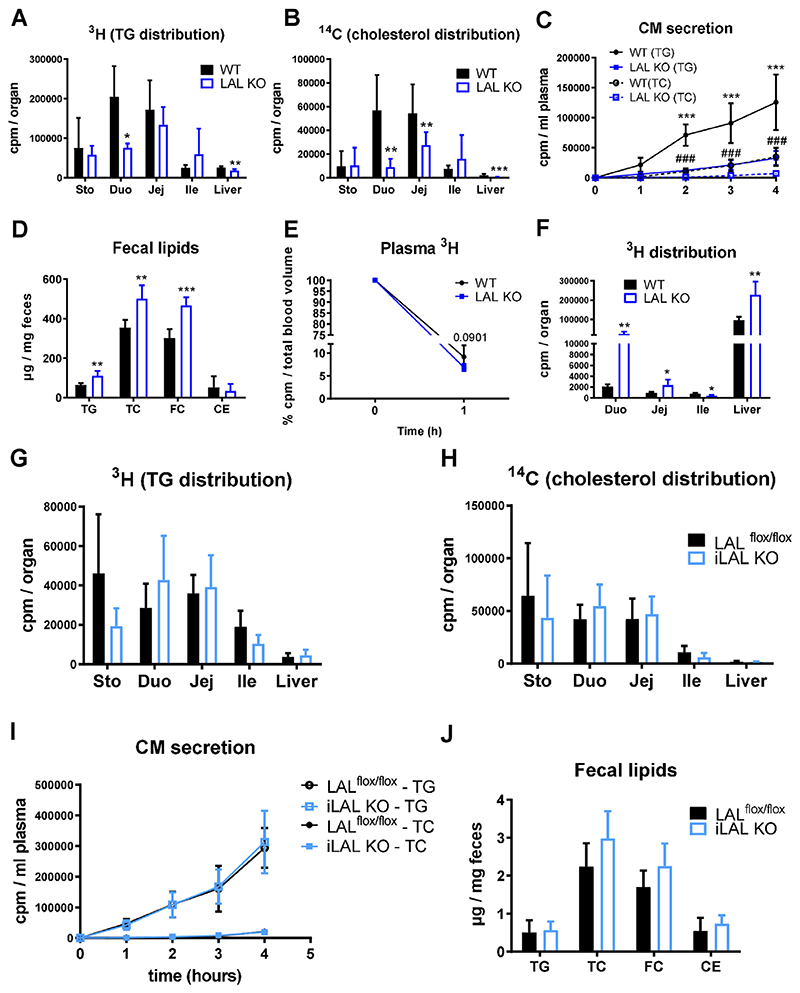
Global but not enterocyte-specific loss of LAL results in decreased dietary lipid uptake but increased basolateral lipid absorption. **(A)** [^3^H]-triolein and **(B)** [^14^C]- cholesterol distribution in stomach, duodenum, jejunum, ileum, and liver of female LAL KO mice. **(C)** Radioactivity in plasma 1, 2, 3, and 4 h after oral substrate administration. **(D)** Fecal lipid content of female LAL KO mice. Distribution of [^3^H]-triolein-labeled VLDL in **(E)** plasma and **(F)** SI and liver of female LAL KO mice 1 h after i.v. injection. Data represent mean (n = 4–5) + SD; *p < 0.05, **p ≤ 0.01, ***p ≤ 0.001; ###p ≤ 0.001 relative to CM secretion in WT(TC). **(G)** [^3^H]-triolein and **(H)** [^14^C]-cholesterol distribution in stomach, duodenum, jejunum, ileum, and liver of female iLAL KO mice. **(I)** Radioactivity in plasma 1, 2, 3, and 4 h after oral substrate administration. **(J)** Fecal lipid content of male iLAL KO mice. Data represent mean (n = 3–5) + SD.

We next examined whether global LAL-D affected lipid absorption from the diet and subsequent CM secretion or lipid uptake from the circulation at the basolateral side of enterocytes. To this end, we gavaged mice with corn oil supplemented with 0.2% cholesterol, [^3^H]-triolein, and [^14^C]-cholesterol after an intraperitoneal injection of poloxamer P-407 to block peripheral lipolysis. In LAL KO mice, both intestinal TG (Figure 4A) and cholesterol (Figure 4B) absorption were markedly delayed compared to WT mice, which in turn resulted in a lower CM secretion rate (Figure 4C) and, ultimately, in less appearance of the radioactive tracer in the liver (Figure 4A,B). These results, together with an increased fecal lipid excretion (Figure 4D), indicated impaired dietary lipid absorption in LAL KO mice.

Given that the SI can absorb lipids from the circulation in addition to lipids from the diet, we investigated whether the observed intestinal lipid accumulation in LAL KO mice was due to increased basolateral uptake of circulating lipoproteins. We therefore injected mice i.v. with human [^3^H]-triolein-VLDL. After 1 h, LAL KO mice showed a slightly accelerated clearance of the radioactive tracer from the circulation compared to WT mice (Figure 4E) but much higher substrate uptake in the proximal intestine (Figure 4F). The deposition of the tracer predominantly in the duodenum of LAL KO mice was consistent with the massive lipid accumulation observed histologically. Of note, basolaterally-administered lipoproteins also accumulated in the liver of LAL KO mice. Conversely, iLAL KO mice showed unaltered lipid absorption and CM secretion (Figure 4G-J), confirming the negligible role of enterocyte LAL activity in the phenotype of global LAL deficiency.

In summary, these results could explain the enormous lipid accumulation in infiltrating macrophages but not in enterocytes, since macrophages are the first cell type to come into contact with circulating lipoproteins after they have left the blood vessel in the lamina propria. Additionally, these experiments may explain, at least in part, why fatty macrophages are predominantly present in the duodenum and less so in the distal intestine.

### 3.5 Global but not intestinal LAL deficiency affects intestinal lipid metabolism and inflammation

**Figure 5:**
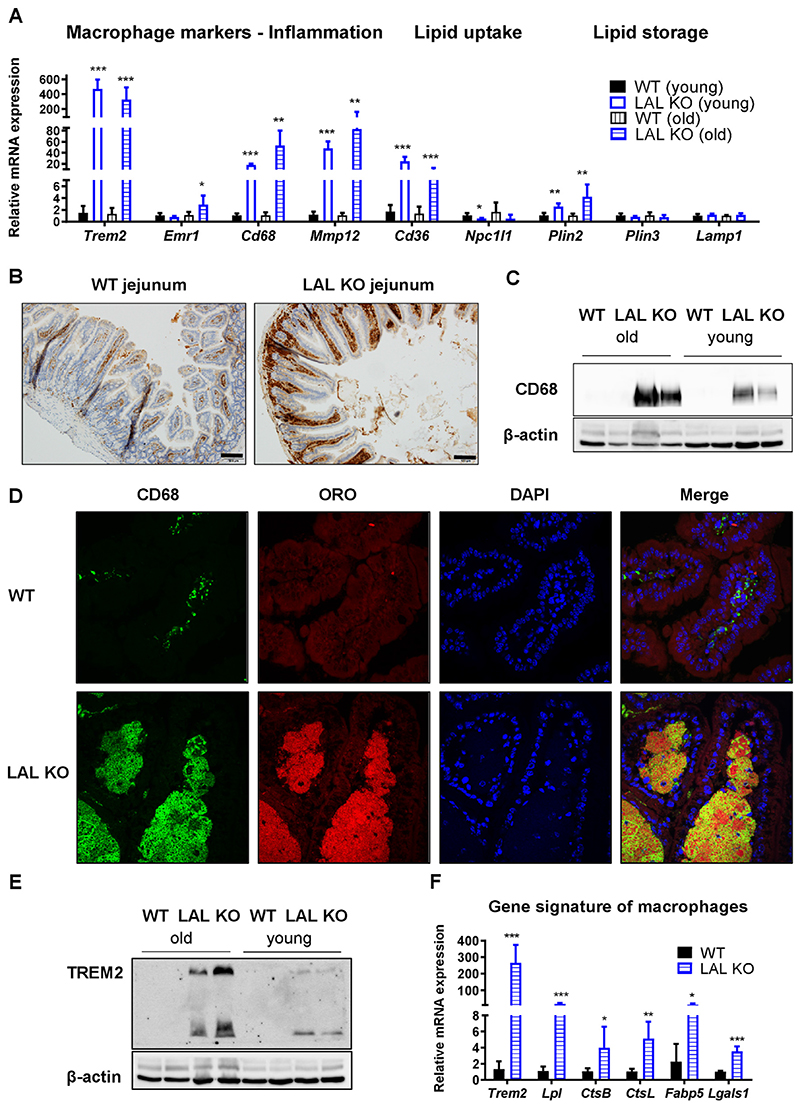
Lipid-associated macrophages-like gene signature in the duodenum of LAL KO mice. **(A)** mRNA expression of various inflammatory and lipid metabolism-related genes in duodena of chow diet-fed young (8–14 weeks) and old (40–50 weeks) LAL KO mice (n = 4–5). **(B)** CD68 IHC staining of WT and LAL KO jejuna. Scale bar, 100 μm. Duodenal protein expression of **(C)** CD68 and **(E)** TREM2 in young and old WT and LAL KO mice. **(D)** CD68 IHC with oil red O and DAPI co-staining to visualize macrophages (green), neutral lipids (red), and nuclei (blue), respectively, in duodenal sections of WT and LAL KO mice. **(F)** Lipid-associated macrophages-like gene signature of macrophages in duodena of old LAL KO mice (n = 4–5). Data represent mean values + SD; *p < 0.05, **p ≤ 0.01, ***p ≤ 0.001.

As LAL-D worsens with age in mice [27], we analyzed the mRNA expression levels of several genes related to lipid metabolism (e.g. transporters, lipid droplet-associated proteins) and inflammation (e.g. macrophage markers) in young (8–14 weeks) and old (40–50 weeks) LAL KO mice. Gene expression in the duodenum, the most affected part of the SI, was differentially altered between young and old LAL KO mice (Figure 5A). Of note, macrophage markers and genes involved in lipid uptake and storage were even higher increased in the jejunum of old LAL KO mice (Supplementary Fig. S3A), confirming that the phenotype of LAL-D deteriorates with age. Since enterocyte-specific LAL-D did not result in the drastic intestinal phenotype observed in mice globally lacking LAL, we examined whether a compensatory upregulation might be present in iLAL KO mice. However, intestinal gene expression levels were comparable to control mice, irrespective of diet, age, or part of the SI (Supplementary Figs. S3B and C).

To investigate the origin of immune cell invasion into the lamina propria in LAL KO mice, we performed IHC against CD68, a well-known marker for circulating macrophages [41,42]. Although the intensity of staining in the duodenum of LAL KO mice was not as pronounced (Supplementary Fig. S3D), probably due to the excessive amount of lipids, we observed more CD68-positive cells (Figure 5B) and confirmed the increased CD68 protein expression in duodena of old versus young LAL KO mice (Figure 5C), consistent with qPCR results (Figure 5A). In addition, we performed DAPI staining, CD68 immunohistochemistry, and ORO staining in duodenal sections of WT and LAL KO mice. We observed numerous CD68-positive cells in LAL KO mice, and ORO staining revealed the presence of large amounts of neutral lipids. The merged image shows co-localization of CD68-positive cells and ORO-positive lipids, confirming that macrophages are the cells harboring the lipid infiltrates (Figure 5D). Given the massive increase in Trem2 gene and protein expression (Figure 5A,E), we finally assessed whether these macrophages were comparable to the recently described macrophage subclass of lipid-associated macrophages (LAMs), which have been attributed a preventive role against systemic hypercholesterolemia and adipocyte hypertrophy [43]. We therefore analyzed the gene expression signature assigned to LAMs, such as *Trem2, Lipa, Lpl, Ctsb, Ctsl, Fabp4, Fabp5, Lgals1, Lgals3, Cd9*, and *Cd36* [43]. We found that the expression of most of these genes was highly increased (Figure 5F), suggesting that macrophages infiltrating the lamina propria of LAL KO mice have a comparable gene signature to LAMs and “disease-associated microglia” (DAM) identified in an Alzheimer's disease model [44].

## 4 Discussion

Intestinal epithelial cells (enterocytes) are capable of absorbing dietary fat, packaging it into CMs, and secreting them upon need. This makes enterocytes one of the main sources of lipids to meet the energy demands of an organism. In its severe form in infancy, LAL-D leads to a lethal condition with massive accumulation of CEs and TGs in various tissues, including liver, spleen, macrophages, and SI [32]. As lipid malabsorption is one of the major symptoms of LAL-D patients [19], we aimed to elucidate the role of LAL in the SI and the interplay between enterocytes and macrophages using global and enterocyte-specific LAL KO mouse models.

The increased number of immune cells, especially macrophages, in various organs such as liver, lung or SI of LAL KO mice already suggested that they play a crucial role in the pathogenesis of LAL-D [32]. Our present findings demonstrate that infiltrating macrophages are responsible for the severe lipid accumulation in LAL KO mice, pre-dominantly incorporating lipids from the circulation, as recently suggested [31]. We have identified a macrophage subclass that is very similar to LAMs and DAM and whose most prominent marker is TREM2 [43,45]. TREM2 is a transmembrane protein expressed by monocytic myeloid cells and is essential for the proper function of microglia in Alzheimer's disease and of macrophages in adipose tissue or liver under obese conditions [43,45]. In particular, LAMs are recruited from the circulation to the adipose tissue based on their expression of CD68 to prevent adipocyte hypertrophy and loss of systemic lipid homeostasis in obesity. LAMs possess a conserved gene signature characterized by the expression of genes involved in phagocytosis and lipid metabolism, including *Lipa* [43]. The increased mRNA expression of *Cd68, Trem2*, and other LAM-like signature genes suggests that monocytes are recruited from the circulation to the SI of LAL KO mice, where TREM2, along with the inflammatory environment, drives this gene expression profile to prevent exacerbation of metabolic derangements through their LAM-associated functions. However, since they constitutively lack LAL, they are unable to hydrolyze lipids entrapped in lysosomes, resulting in the accumulation of lipid-filled macrophages in the *lamina propria*. Considering that none of these changes occur in iLAL KO mice, we conclude that LAMs are not recruited to the SI of these mice because the resident macrophages have functional LAL and are able to degrade incoming lipids.

As already shown in previous publications [27,46,47], lipid accumulation in the SI of LAL KO mice increases progressively with age. The number of infiltrating macrophages follows the same pattern, again suggesting that they are crucial players in disease progression and lipid accumulation in the SI of these mice. Macrophage infiltration and tissue lipid accretion in LAL KO tissues was accompanied by BMP accumulation, which is frequently observed in lysosomal storage disorders [48,49]. This negatively charged lipid is a major component of luminal membranes in acidic organelles [50] and acts as a co-factor for lysosomal lipid hydrolases and lipid-binding/transport proteins. In this way, BMP promotes lysosomal lipid degradation and export [51]. In contrast to the strong increase in BMP content in the SI of global LAL KO mice, we did not observe BMP accumulation in the SI of iLAL KO mice, which argues against lysosomal dysfunction and increased lysosome biogenesis in these mice.

Since macrophages of the *lamina propria* are located in close proximity to blood vessels, lipids taken up from the basolateral side (circulation) are more likely to contribute to the formation of lipid-filled macrophages than dietary lipids. Consistent with our data from chow diet-fed LAL KO mice, LAL KO mice fed a Western-type diet had increased basolateral lipid uptake, which was associated with impaired dietary lipid absorption and higher fecal lipid loss [31]. Lipid-filled macrophages are mainly present in the duodenum of LAL KO mice, which is in agreement with the fact that basolateral lipid uptake is more pronounced in the proximal part of the SI and gradually decreases in the jejunum and ileum [23,31,52]. The reduced dietary lipid absorption and increased fecal lipid loss might be a compensatory mechanism of the LAL KO SI to limit the stress of lipid accumulation, independent of the diet.

Despite the high expression of LAL in the intestine of WT mice [53] and the complete loss of *Lipa* expression and LAL activity in enterocytes of iLAL KO mice, these animals did not develop any obvious pathological features. iLAL KO mice exhibited unchanged plasma and intestinal lipid levels and villous morphology. They also showed no signs of atypical lipid-rich macrophage infiltration, independent of age and diet. These results raise the question of whether dietary lipids are destined for lysosomal degradation, and if so, whether they may be degraded by an alternative pathway. An interesting discovery several years ago showed that in Niemann-Pick disease type C, an increased release of exosomes may serve as a protective mechanism against excessive accumulation of lysosomal cholesterol [54]. Exosomes are small vesicles of 50–100 nm that originate from the intraluminal membranes of multivesicular bodies. These vesicles can then be shuttled to the lysosome for degradation or released into the extracellular space [55]. One might speculate that a similar mechanism occurs in the enterocytes of LAL KO mice to prevent excessive accumulation of lysosomal lipids.

In summary, our study shows that loss of LAL exclusively in enter-ocytes is not a sufficient trigger for the intestinal phenotype of global LAL KO mice. Based on the massive lipid accumulation in the intestinal macrophages of LAL KO mice, we conclude that LAL deficiency in infiltrating macrophages and not in enterocytes is responsible for the pathological phenotype of global LAL KO mice.

## Supplementary Material

Fig. S1-S3, Table S1

## Data Availability

Data will be made available on request.

## References

[1] Hamosh M (1990). Lingual and gastric lipases. Nutr Burbank Los Angel Cty Calif.

[2] Lowe ME (1997). Structure and function of pancreatic lipase and colipase. Annu Rev Nutr.

[3] Hui DY (1996). Molecular biology of enzymes involved with cholesterol ester hydrolysis in mammalian tissues. Biochim Biophys Acta BBA - Lipids Lipid Metab.

[4] Friedman HI, Nylund B (1980). Intestinal fat digestion, absorption, and transport A review. Am J Clin Nutr.

[5] Iqbal J, Hussain MM (2009). Intestinal lipid absorption. Endocrinol Metab.

[6] Pan X, Hussain MM (2012). Gut Triglyceride Production. Biochim Biophys Acta.

[7] Levy E, Spahis S, Sinnett D, Peretti N, Maupas-Schwalm F, Delvin E (2007). Intestinal cholesterol transport proteins: an update and beyond. Curr Opin Lipidol.

[8] Hussain MM (2014). Intestinal lipid absorption and lipoprotein formation. Curr Opin Lipidol.

[9] Gordon DA, Jamil H (2000). Progress towards understanding the role of microsomal triglyceride transfer protein in apolipoprotein-B lipoprotein assembly. Biochim Biophys Acta BBA - Mol Cell Biol Lipids.

[10] Robertson MD, Parkes M, Warren BF, Ferguson DJP, Jackson KG, Jewell DP (2003). Mobilisation of enterocyte fat stores by oral glucose in humans. Gut.

[11] Zechner R, Madeo F, Kratky D (2017). Cytosolic lipolysis and lipophagy: two sides of the same coin. Nat Rev Mol Cell Biol.

[12] Singh R, Cuervo AM (2012). Lipophagy: connecting autophagy and lipid metabolism Int. J Cell Biol.

[13] Warner TG, Dambach LM, Shin JH, O'Brien JS (1981). Purification of the lysosomal acid lipase from human liver and its role in lysosomal lipid hydrolysis. J Biol Chem.

[14] Ouimet M, Franklin V, Mak E, Liao X, Tabas I, Marcel YL (2011). Autophagy regulates cholesterol efflux from macrophage foam cells via lysosomal acid lipase. Cell Metabol.

[15] Grumet L, Eichmann TO, Taschler U, Zierler KA, Leopold C, Moustafa T (2016). Lysosomal acid lipase hydrolyzes retinyl ester and affects retinoid turnover. J Biol Chem.

[16] Khaldoun SA, Emond-Boisjoly M-A, Chateau D, Carrière V, Lacasa M, Rousset M (2014). Autophagosomes contribute to intracellular lipid distribution in enterocytes. Mol Biol Cell.

[17] Seok S, Kim Y-C, Zhang Y, Kong B, Guo G, Ma J (2022). Feeding activates FGF15-SHP-TFEB-mediated lipophagy in the gut. EMBO J.

[18] Bernstein DL, Hülkova H, Bialer MG, Desnick RJ (2013). Cholesteryl ester storage disease: review of the findings in 135 reported patients with an underdiagnosed disease. J Hepatol.

[19] Pericleous M, Kelly C, Wang T, Livingstone C, Ala A (2017). Wolman's disease and cholesteryl ester storage disorder: the phenotypic spectrum of lysosomal acid lipase deficiency. Lancet Gastroenterol Hepatol.

[20] Boldrini R, Devito R, Biselli R, Filocamo M, Bosman C (2004). Wolman disease and cholesteryl ester storage disease diagnosed by histological and ultrastructural examination of intestinal and liver biopsy. Pathol Res Pract.

[21] Leopold C, Duta-Mare M, Sachdev V, Goeritzer M, Maresch LK, Kolb D (2019). Hepatocyte-specific lysosomal acid lipase deficiency protects mice from diet-induced obesity but promotes hepatic inflammation. Biochim Biophys Acta Mol Cell Biol Lipids.

[22] Madison BB, Dunbar L, Qiao XT, Braunstein K, Braunstein E, Gumucio DL (2002). Cis elements of the villin gene control expression in restricted domains of the vertical (crypt) and horizontal (duodenum, cecum) axes of the intestine. J Biol Chem.

[23] Korbelius M, Vujic N, Sachdev V, Obrowsky S, Rainer S, Gottschalk B (2019). ATGL/CGI-58-Dependent hydrolysis of a lipid storage pool in murine enter-ocytes. Cell Rep.

[24] Fischer AH, Jacobson KA, Rose J, Zeller R Hematoxylin and eosin staining of tissue and cell sections. Cold Spring Harb Protoc.

[25] Vujic N, Korbelius M, Leopold C, Duta-Mare M, Rainer S, Schlager S (2017). Monoglyceride lipase deficiency affects hepatic cholesterol metabolism and lipid-dependent gut transit in ApoE-/- mice. Oncotarget.

[26] Khalifeh-Soltani A, Gupta D, Ha A, Iqbal J, Hussain M, Podolsky MJ Mfge8 regulates enterocyte lipid storage by promoting enterocyte triglyceride hydrolase activity. JCI Insight.

[27] Kuentzel KB, Bradic I, Akhmetshina A, Korbelius M, Rainer S, Kolb D (2021). Defective lysosomal lipolysis causes prenatal lipid accumulation and exacerbates immediately after birth. Int J Mol Sci.

[28] Schweiger M, Eichmann TO, Taschler U, Zimmermann R, Zechner R, Lass A (2014). Methods Enzymol.

[29] Mastronarde DNSerialEM (2003). A program for automated tilt series acquisition on Tecnai microscopes using prediction of specimen position. Microsc Microanal.

[30] Matyash V, Liebisch G, Kurzchalia TV, Shevchenko A, Schwudke D (2008). Lipid extraction by methyl-tert-butyl ether for high-throughput lipidomics. J Lipid Res.

[31] Sachdev V, Duta-Mare M, Korbelius M, Vujic N, Leopold C, Freark de Boer J (2021). Impaired bile acid metabolism and gut dysbiosis in mice lacking lysosomal acid lipase. Cells.

[32] Du H, Heur M, Duanmu M, Grabowski GA, Hui DY, Witte DP (2001). Lysosomal acid lipase-deficient mice: depletion of white and Brown fat, severe hep-atosplenomegaly, and shortened life span. J Lipid Res.

[33] Bradic I, Kuentzel KB, Honeder S, Grabner GF, Vujic N, Zimmermann R (2022). Off-target effects of the lysosomal acid lipase inhibitors lalistat-1 and lalistat-2 on neutral lipid hydrolases. Mol Metabol.

[34] Radovic B, Vujic N, Leopold C, Schlager S, Goeritzer M, Patankar JV (2016). Lysosomal acid lipase regulates VLDL synthesis and insulin sensitivity in mice. Diabetologia.

[35] Duta-Mare M, Sachdev V, Leopold C, Kolb D, Vujic N, Korbelius M (2018). Lysosomal acid lipase regulates fatty acid channeling in Brown adipose tissue to maintain thermogenesis. Biochim Biophys Acta.

[36] Kobayashi T, Beuchat M-H, Chevallier J, Makino A, Mayran N, Escola J-M (2002). Separation and characterization of late endosomal membrane domains. J Biol Chem.

[37] Showalter MR, Berg AL, Nagourney A, Heil H, Carraway KL, Fiehn O (2020). The emerging and diverse roles of bis(monoacylglycero) phosphate lipids in cellular physiology and disease. Int J Mol Sci.

[38] Liu N, Tengstrand EA, Chourb L, Hsieh FY (2014). Di-22:6-Bis(Monoacylglycerol) Phosphate: a clinical biomarker of drug-induced phospholipidosis for drug development and safety assessment. Toxicol Appl Pharmacol.

[39] Hein LK, Duplock S, Fuller M (2013). Selective reduction of bis(Monoacylglycero) Phosphate ameliorates the storage burden in a THP-1 macrophage model of gaucher disease. J Lipid Res.

[40] Käkelä R, Somerharju P, Tyynelä J (2003). Analysis of phospholipid molecular species in brains from patients with infantile and juvenile neuronal-ceroid lipofuscinosis using liquid chromatography-electrospray ionization mass spectrometry. J Neurochem.

[41] Holness CL, Simmons DL Molecular cloning of CD68, a human macrophage marker related to lysosomal glycoproteins.

[42] Iqbal AJ, McNeill E, Kapellos TS, Regan-Komito D, Norman S, Burd S (2014). Human CD68 promoter GFP transgenic mice allow analysis of monocyte to macrophage differentiation in vivo. Blood.

[43] Jaitin DA, Adlung L, Thaiss CA, Weiner A, Li B, Descamps H (2019). Lipid-associated macrophages control metabolic homeostasis in a trem2-dependent manner. Cell.

[44] Keren-Shaul H, Spinrad A, Weiner A, Matcovitch-Natan O, Dvir-Szternfeld R, Ulland TK (2017). A unique microglia type associated with restricting development of Alzheimer's disease. Cell.

[45] Wang Y, Cella M, Mallinson K, Ulrich JD, Young KL, Robinette ML (2015). TREM2 lipid sensing sustains the microglial response in an Alzheimer's disease model. Cell.

[46] Lopez AM, Posey KS, Turley SD (2014). Deletion of sterol O-acyltransferase 2 (SOAT2) function in mice deficient in lysosomal acid lipase (LAL) dramatically reduces esterified cholesterol sequestration in the small intestine and liver. Biochem Biophys Res Commun.

[47] Aqul A, Lopez AM, Posey KS, Taylor AM, Repa JJ, Burns DK (2014). Hepatic entrapment of esterified cholesterol drives continual expansion of whole body sterol pool in lysosomal acid lipase-deficient mice. Am J Physiol Gastrointest Liver Physiol.

[48] Meikle PJ, Duplock S, Blacklock D, Whitfield PD, Macintosh G, Hopwood JJ (2008). Effect of lysosomal storage on bis(Monoacylglycero)Phosphate. Biochem J.

[49] Grabner GF, Fawzy N, Pribasnig MA, Trieb M, Taschler U, Holzer M (2019). Metabolic disease and ABHD6 alter the circulating bis(Monoacylglycerol) Phosphate profile in mice and humans. J Lipid Res.

[50] Hullin-Matsuda F, Luquain-Costaz C, Bouvier J, Delton-Vandenbroucke I (2009). Bis(Monoacylglycero)Phosphate, a peculiar phospholipid to control the fate of cholesterol: implications in pathology. Prostaglandins Leukot Essent Fatty Acids.

[51] Gallala HD, Sandhoff K (2011). Biological function of the cellular lipid BMP—BMP as a key activator for cholesterol sorting and membrane digestion. Neurochem Res.

[52] Li D, Rodia CN, Johnson ZK, Bae M, Muter A, Heussinger AE (2019). Intestinal basolateral lipid substrate transport is linked to chylomicron secretion and is regulated by ApoC-III. J Lipid Res.

[53] Du H, Witte DP, Grabowski GA (1996). Tissue and cellular specific expression of murine lysosomal acid lipase MRNA and protein. J Lipid Res.

[54] Strauss K, Goebel C, Runz H, Möbius W, Weiss S, Feussner I (2010). Exosome secretion ameliorates lysosomal storage of cholesterol in niemann-pick type C disease. J Biol Chem.

[55] Keller S, Sanderson MP, Stoeck A, Altevogt P (2006). Exosomes: from biogenesis and secretion to biological function. Immunol Lett.

