# Supplementary material for: Impact of (intestinal) LAL deficiency on lipid metabolism and macrophage infiltration
Source: Mol Metab. Author manuscript; Available in PMC 2023 Jun 1. (PMC10209539; doi:10.1016/j.molmet.2023.101737)
Supplement: Fig. S1-S3, Table S1 [file EMS176369-supplement-Fig__S1_S3__Table_S1.pdf]

Supplemental information

Impact of (intestinal) LAL deficiency on lipid metabolism and macrophage infiltration

Valentina Bianco, Melanie Korbelius, Nemanja Vujic, Alena Akhmetshina, Melina Amor, Dagmar Kolb, Anita Pirchheim, Ivan Bradic, Katharina B. Kuentzel, Martin Buerger, Silvia Schauer, Huyen T.T. Phan, Dominik Bulfon, Gerald Hoefler, Robert Zimmermann, and Dagmar Kratky

Figure S1

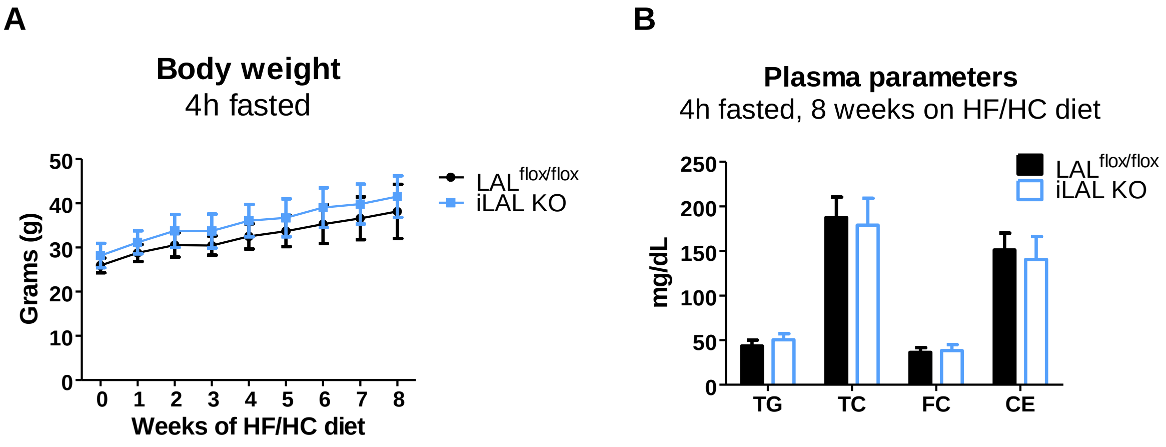

Figure S1: Unchanged body weight and plasma lipid parameters in HF/HCD-fed iLAL KO mice. (A) Body weight gain during and **(B)** plasma lipid parameters after 8 weeks of HF/HCD after 4 h of fasting in male iLAL KO and control littermates (n=5-7). Data represent mean + SD.

Figure S2

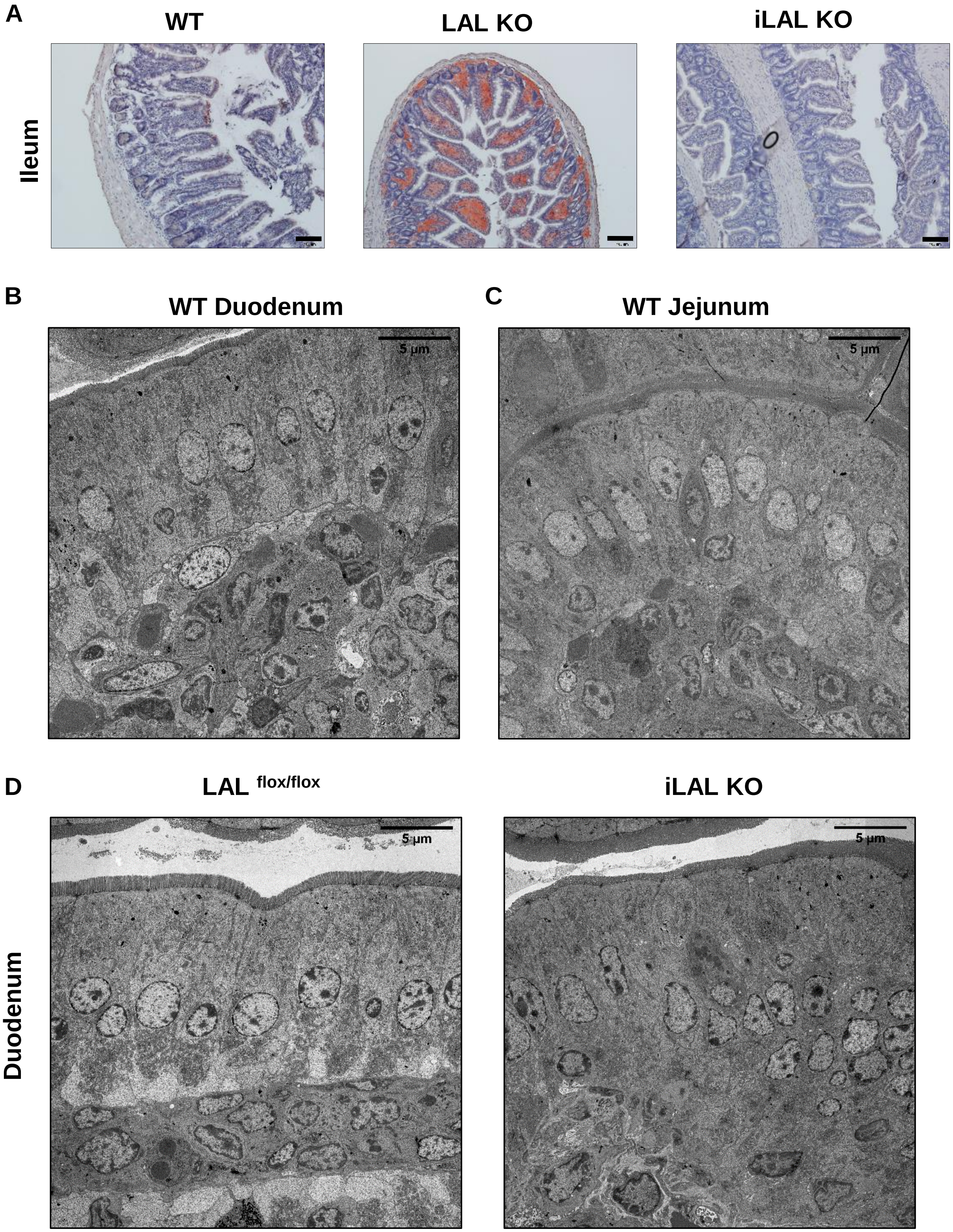
A
WT
LAL KO
iLAL KO
Ileum
B
WT Duodenum
5 µm
C
WT Jejunum
5 µm
D
LAL flox/flox
iLAL KO
Duodenum
5 µm
5 µm

E

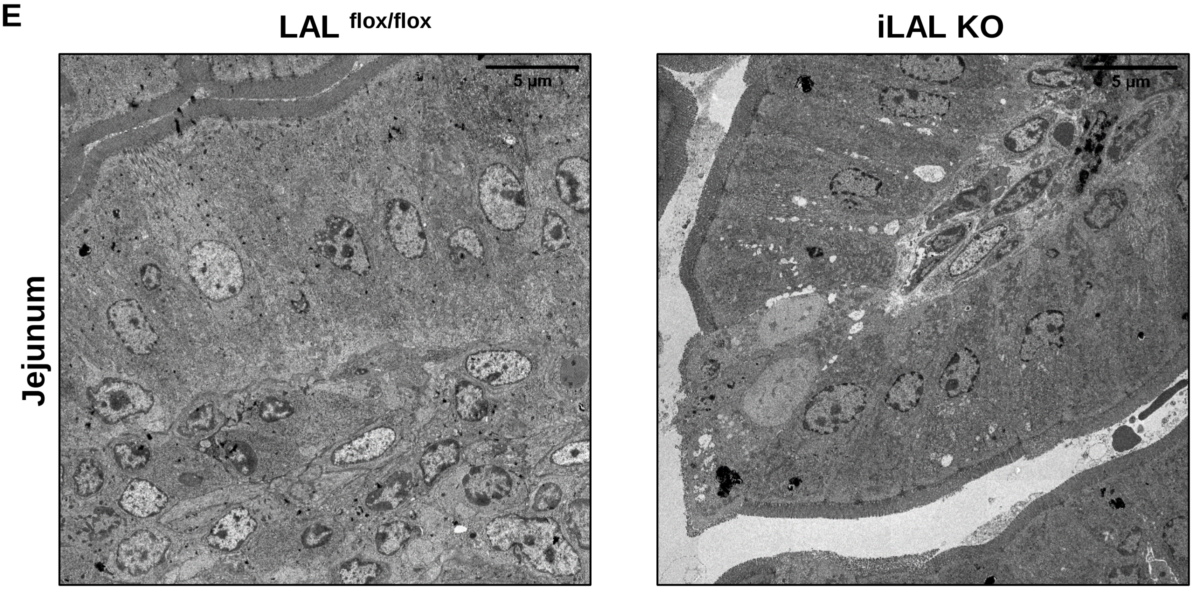

F

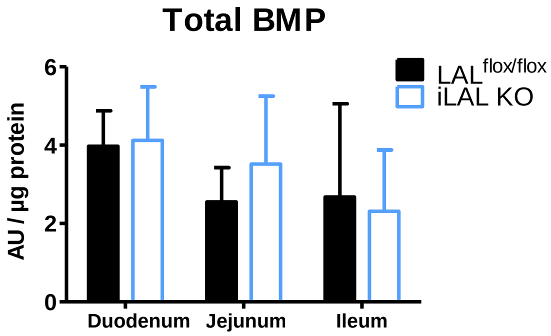

Figure S2: Unchanged lipid accumulation and BMP content in iLAL KO mice. (A) Oil **r**ed O staining of jejunal and ileal sections from 6-h fasted WT, LAL KO, and iLAL KO mice (scale bar, 100 µm). Electron micrographs of **(B)** duodenal and **(C)** jejunal sections from male WT mice as well as of **(D)** duodenal and **(E)** jejunal sections from male LAL$^{flox/flox}$ and iLAL KO mice. Scale bar, 10 µm. **(F)** BMP quantification in duodenal, jejunal, and ileal scrapings of female iLAL KO mice (n=4-5). Data represent mean + SD.

Figure S3

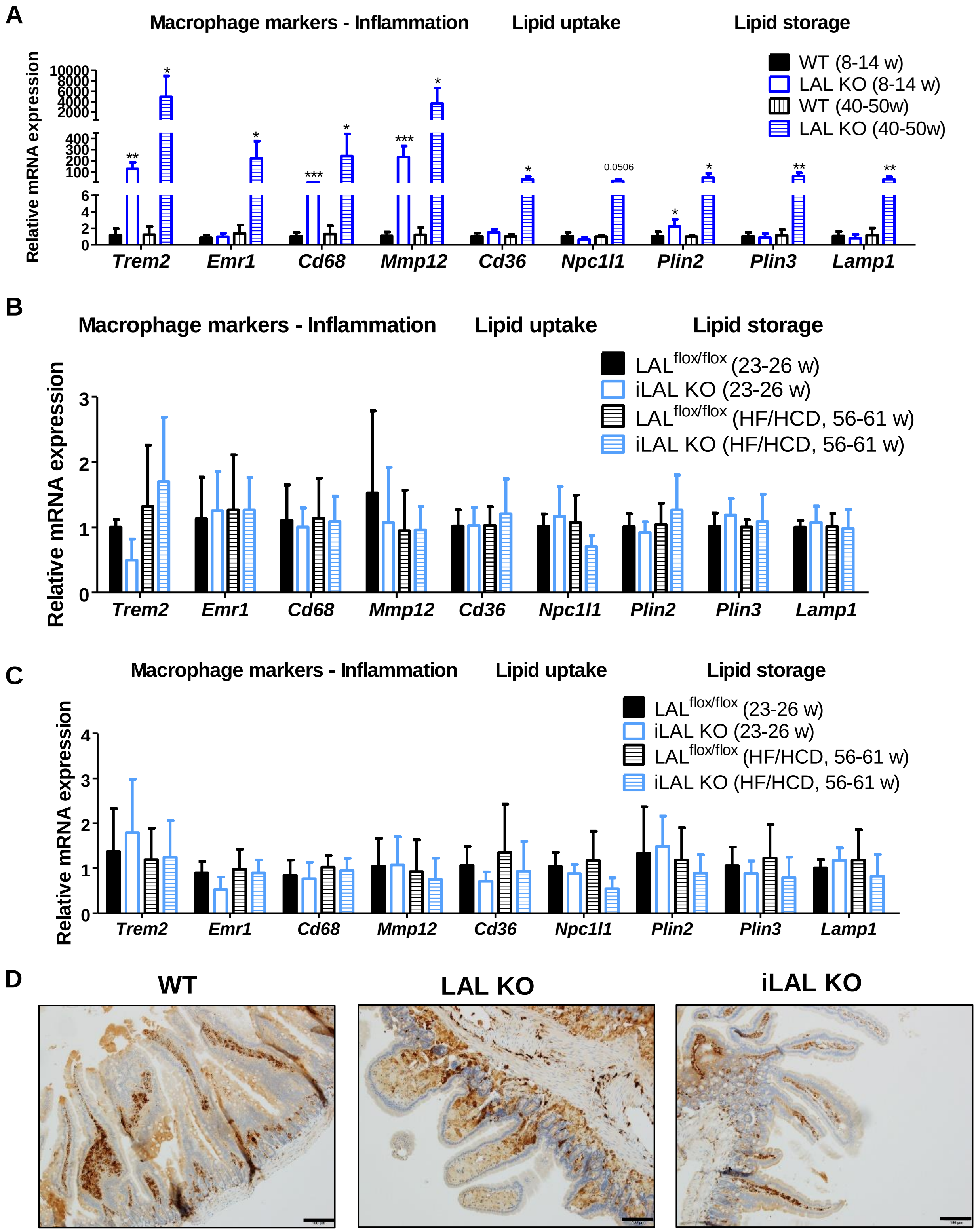

Figure S3: Unchanged gene expression profile in iLAL KO mice. mRNA expression of inflammation and lipid metabolism-related genes in **(A)** jejunum of young and old LAL KO mice, **(B)** duodenum and **(C)** jejunum of chow- and 8-week HF/HCD-fed iLAL KO mice. **(D)** CD68 IHC staining of WT, LAL KO, and iLAL KO duodena. Scale bar, 100 µm. Data represent mean (n=3-7) + SD. *p<0.05, **p≤0.01, *** p≤0.001.

Table S1: Primer sequences

Gene	Forward Primer	Reverse Primer
Cd36	GCAGGTCTATCTACGCTGTG	GGTTGTCTGGATTCTGGAGG
Cd68	AACAGGACCTACATCAGAGC	TCAAGGTGAACAGCTGGAGA
CtsB	TTGCGTTCGGTGAGGACATAG	GCAGGAGCCCTGGTCTCTA
CtsL	ACAGAAGACTGTATGGCACGA	GTATTCCCCGTTGTGTAGCTG
Emr1	CTTTGGCTATGGGCTTCCAGTC	GCAAGGAGGACAGAGTTTATCGTG
Fabp5	TGACCCTCATGGAAGGTTAGAA	GGACATTGCATTGCATGTTGG
Lipa	GCTGGCTTTGATGTGTGGATG	ATGGTGCAGCCTTGAGAATGA
Lamp1	CAGCACTCTTTGAGGTGAAAAAC	CCATTCGCAGTCTCGTAGGTG
Lgals1	CAAGCTGCCAGACGGACAT	AGGCCACGCACTTAATCTTGA
Lpl	ACATTCCCGTTACCGTCCATC	GGACCCCTGAAGACACAG
Mmp12	CTGCTCCCATGAATGACAGTG	AGTTGCTTCTAGCCCAAAGAAC
Npc1l1	TGTCCCCGCCTTATACAATGG	CCTTGGTGATAGACAGGCTACTG
Plin2	CTTGTGTCCTCCGCTTATGCT	GCAGAGGTCACGGTCTTCAC
Plin3	ATGTCTAGCAATGGTACAGATGC	CGTGGAACTGATAAGAGGCAGG
Trem2	CTGGAACCGTCACCATCACTC	CGAAACTCGATGACTCCTCGG
